# Temperature Effects on the Propagation Characteristics of Love Waves along Multi-Guide Layers of Sio_2_/Su-8 on St-90°X Quartz

**DOI:** 10.3390/s120607337

**Published:** 2012-05-30

**Authors:** Fangqian Xu, Wen Wang, Jiaoli Hou, Minghua Liu

**Affiliations:** 1 Zhejiang University of Media and Communications, Hangzhou 310018, China; E-Mail: xufangqian2005@163.com; 2 Institute of Acoustics, Chinese Academy of Sciences, Beijing 100190, China; E-Mails: hou720@yahoo.com.cn (J.H.); liuminghua@mail.ioa.ac.cn (M.L.)

**Keywords:** Love wave, multi-guide, SU-8, SiO_2_, ST-90°X quartz, *Tcf*

## Abstract

Theoretical calculations have been performed on the temperature effects on the propagation characteristics of Love waves in layered structures by solving the coupled electromechanical field equations, and the optimal design parameters were extracted for temperature stability improvement. Based on the theoretical analysis, excellent temperature coefficient of frequency (*Tcf*) of the fabricated Love wave devices with guide layers of SU-8/SiO_2_ on ST-90°X quartz substrate is evaluated experimentally as only 2.16 ppm.

## 1. Introduction

Recently, there has been great interest in Love wave devices in bio or chemical sensors owing to their very low longitudinal coupling attenuation, higher mass loading sensitivity and effective interdigital transducers (IDTs) protection in harsh gas and liquid environments [1–5]. Typical Love wave devices are composed of a piezoelectric substrate with an IDT pattern supporting a shear horizontal surface acoustic wave (SH-SAW), and a thin waveguide layer on the top of the substrate. Due to the waveguide effect, the acoustic energy is trapped into the thin guide layer, resulting in larger mass loading effects from any applied perturbation. One of the conditions for Love wave formation is the SH-SAW with high shear velocity propagating along the piezoelectric substrates. Another condition for the existence of Love wave mode is that the shear velocity in the guiding layer is smaller than the shear velocity in the substrate. As the difference of the shear velocities between the substrate and guiding layer becomes larger, the conversion efficiency of acoustic energy into the Love wave is increased, resulting in higher sensitivity to applied perturbations.

Various dielectric materials such as silicon dioxide (SiO_2_) and polymers can be used as the waveguide materials [6]. SiO_2_ has been widely used for Love wave sensors because it presents the advantages of good rigidity, low acoustic loss, and high mechanical and chemical resistance. Nevertheless, the polymers have some advantages over SiO2 for Love wave sensor implementation because they are more efficient than SiO_2_ in converting the bulk SH mode to the Love wave mode due to their lower shear bulk velocity and lower density as compared to that of SiO_2_, resulting in an order of magnitude improvement in mass sensitivity [7]. Also, they are easier to deposit onto the substrate than SiO_2_.

Recently, a new Love wave structure containing both SiO_2_ and polymer films as a multilayer waveguide was proposed, and some promising results in the form of higher mass loading sensitivity and superior temperature stability were reported. Du *et al.* presented a new Love wave structure consisting of PMMA/SiO2/ST-quartz, which aims to utilize the merits of both PMMA and SiO2 and improve the overall performance of the devices [8,9]. Using the reversed polarity of the *Tcf* values of the materials for guide layer and substrate listed in Table 1, it is possible to realize a significant reduction in the temperature coefficients of the hybrid device, resulting in improvement of the temperature stability. The SiO_2_ thin film was frequently reported as the over-layer on LiNbO_3_ substrates to improve the temperature stability due to its reverse polarity temperature coefficient for the substrate, and related theoretical calculation and experiment were presented in some literatures [10–13]. However, to our knowledge, despite some meaningful performance of Love waves in multi-layered structures, there is still no systematic theoretical study dealing with the propagation properties of Love wave along multi-guide layers, and the effects on temperature stability of the thickness of the guide layers.

**Figure 1.**
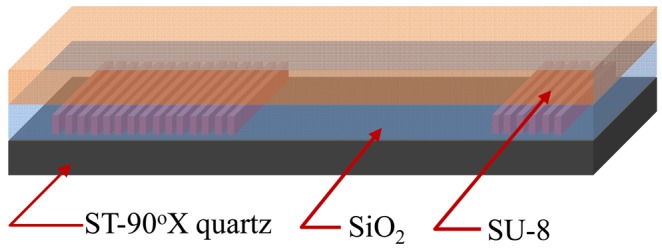
The schematic of the Love wave device with multi-guide layers of SiO_2_/SU-8.

The purpose of this paper is to describes the Love wave propagation along the ST-90°X quartz substrate using a multi-guide structure like SiO_2_/SU-8, as shown in Figure 1. A SU-8 thin layer is proposed as guide layer, which exhibits low shear SH velocity, and benefits for the improvement of behavior and durability of a Love-type device when working as a viscosity sensor in liquid media [14,15].

A theoretical model was established to extract the optimal design parameters of the Love wave device with multi-guides by solving the coupled electromechanical field equation [11], and the temperature coefficient of frequency (Tcf) of such a Love wave structure was calculated theoretically. Then the theoretical results were confirmed by the experimental results, in which, a Love wave delay line with operation frequency of 174 MHz and SiO_2_/SU-8 guiding layer on ST-90°X quartz substrate was fabricated. Very excellent temperature stability of ∼2.16 ppm was observed from the fabricated Love wave device in case where 0.21 μm SiO_2_ and 0.8 μm SU-8 were coated as the guide layers.

## 2. Theoretical Analysis

### 2.1. Theory Model

**Figure 2.**
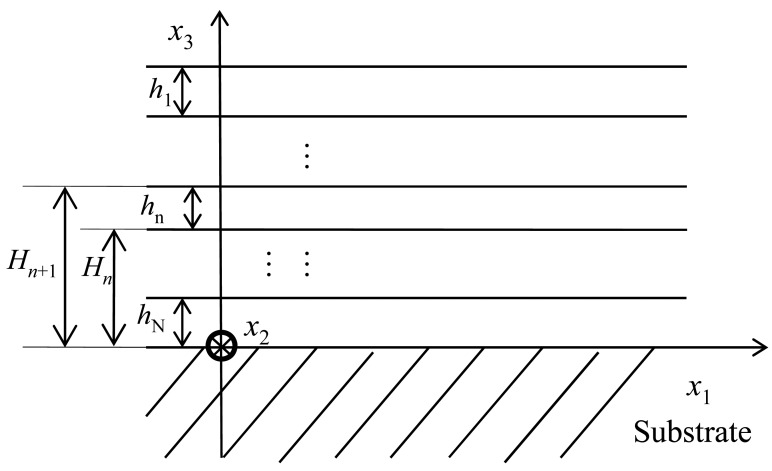
The coordination system in this study.

In this section, the temperature coefficient of frequency (*Tcf*) of a Love wave device with multi-guide layers was analyzed by solving the coupled electromechanical field equation combing with the approximate method described by Tomar *et al.* [11]. For the theoretical approach of the Love wave device, a layered structure composed of a semi-infinite piezoelectric substrate with IDT pattern, and multi-guide layers was constructed, as shown in Figure 2. In the present work the theoretical calculations on the layered structures is an application of the theory of elastic wave propagation developed by Farnell and Adler [16–18].

The coordinate system for the theoretical analysis of Love wave propagation along the layered structure is shown in Figure 2. All the parameters of the media are transformed into this coordinate system. It is well known that the constitutive equations in a piezoelectric medium are as follows:

(1) T=c:S−e'⋅E

(2) D=ɛ⋅E+e:S

where **c, e**, and **ε** represent the elastic, piezoelectric, and permittivity constants matrices. **e'** is the transposition of **e**. The vectors **D** and **E** denote the electric displacement and the electric field intensity. The vectors **T** and **S** indicate the stress tensor and the strain tensor. The strain vector **S** can be expressed with the following term:

(3) $$\text{S}=\nabla_{\text{S}}\cdot \text{u}$$

where ∇_s_ denotes the so-called symmetric gradient operator and the vector u denotes the particle displacement. The quasi-static approximation is adopted, where it is assumed that the electric field intensity can be described by the gradient of a scalar electric potential φ:

(4) E=−∇φ

Equations (3) and (4) can be used to eliminate the vectors **S** and **E** from the constitutive Equations (1) and (2). Gravity and other initial stresses in the medium are neglected, thus the particle motion satisfies Newton's law:

(5) $$\nabla \cdot \text{T}=\rho \frac{\partial^{2}\text{u}}{\partial t^{2}}$$

No external electric charges exist within the medium. Thus, the divergence of the electric displacement is zero:

(6) ∇⋅D=0

For the analysis situation mentioned above, the particle motion and the electric field in such a piezoelectric medium satisfy the following equations:

(7) $$\{\begin{array}{l} c_{\text{ijkl}}\frac{\partial^{2}u_{k}}{\partial x_{j}\partial x_{l}}+e_{\text{ijk}}\frac{\partial^{2}\varphi }{\partial x_{i}\partial x_{k}}-\rho \frac{\partial^{2}u_{k}}{\partial t^{2}}=0\\ e_{\text{jkl}}\frac{\partial^{2}u_{k}}{\partial x_{j}\partial x_{l}}-{ɛ}_{jk}\frac{\partial^{2}\varphi }{\partial x_{j}\partial x_{k}}=0 \end{array}i,j,k,l=1,2,3$$

where ρ is the mass density of the considered material. Equation (7) describes the propagation of the acoustic wave and its associated electric field. The Love wave mode (*u_1_, u_3_*) decoupling with *u_2_* and *φ* while *u_2_* coupling with *φ* is discussed in this paper.

#### (1) In the piezoelectric substrate

The substrate in this paper is a piezoelectric medium whose particle motion in the x_2_-direction is coupled with the electric field. The general solution assumed within the substrate is:

(8) $$\{\begin{array}{c} u_{2}=U\text{exp}\{-ik\left(x_{1}+\beta x_{3}\right)\}\text{exp}(\text{iwt})\\ \varphi =\Phi \text{exp}\{-ik\left(x_{1}+\beta x_{3}\right)\}\text{exp}(\text{iwt}) \end{array}$$

here U and Φ are the magnitudes of the particle motion and the electric potential. β is a deep attenuation factor; ù is an angular frequency; k = 2π/λ is a wave number and λ is a wavelength; $\text{i}=\sqrt{-1}$ is an imaginary unit. In this paper, both k and ù are assumed to be positive, real numbers. Substitution of the general solution Equation (8) into Equation (7) produces a homogeneous algebraic set:

(9) $$\begin{array}{l} \left[\begin{array}{cc} \Gamma_{11} & \Gamma_{12}\\ \Gamma_{12} & \Gamma_{22} \end{array}\right]\times \left[\begin{array}{c} U\\ \Phi \end{array}\right]=0\\ \Gamma_{11}=c_{44}\beta^{2}+2c_{46}\beta +c_{66}-\rho v^{2}\\ \Gamma_{12}=e_{34}\beta^{2}+\left(e_{14}+e_{36}\right)\beta +e_{16}\\ \Gamma_{22}=-({ɛ}_{33}\beta^{2}+2{ɛ}_{13}\beta +{ɛ}_{11}) \end{array}$$

where *v* = *ω/k* is the phase velocity of the acoustic waves. The determinant of the matrix of the coefficients of the set Equation (9) must vanish for nontrivial solutions, thus a quartic equation for β is obtained. When x_3_ →−∞, the particle displacement *u_2_* and the electric potential *φ* must be zero, which means only the two roots with positive real parts are reasonable. The particular solutions for the acoustic wave and the electric field in the substrate are consequently in the following forms:

(10) $$\left\{ \begin{array}{l} u_{2}=[M_{1}A_{1}\exp(-ik\beta_{S1}x_{3})+M_{2}A_{2}\exp(-ik\beta_{S2}x_{3})]\cdot \exp\{i\left(\omega t-kx_{1}\right)\}\\ \varphi =[M_{1}\exp(-ik\beta_{S1}x_{3})+M_{2}\exp(-ik\beta_{S2}x_{3})]\cdot \exp\{i\left(\omega t-kx_{1}\right)\} \end{array}\right.$$

where A is the ratio of U to Φ and:

(11) $$\{\begin{array}{c} A_{1}=\frac{U}{\Phi }{|}_{\beta_{1}}=\frac{{ɛ}_{33}\beta_{S1}^{2}+2{ɛ}_{13}\beta_{S1}+{ɛ}_{11}}{e_{34}\beta_{S1}^{2}+\left(e_{14}+e_{36}\right)\beta_{S1}+e_{16}}\\ A_{2}=\frac{U}{\Phi }{|}_{\beta_{2}}=\frac{{ɛ}_{33}\beta_{S2}^{2}+2{ɛ}_{13}\beta_{S2}+{ɛ}_{11}}{e_{34}\beta_{S2}^{2}+\left(e_{14}+e_{36}\right)\beta_{S2}+e_{16}} \end{array}$$

The M coefficient in Equation (11) and in the following expressions is the coefficient to be determined by the boundary conditions.

#### (2) In the elastic wave-guide layer

The particle displacement is uncoupled from the electric field within the layers because the layers are assumed to be isotropic materials without piezoelectricity. Suppose several layers were overlaid on the substrate, define the top layer the first one, the one above the substrate the N^th^. The layer is finite in the x_3_-direction and the solution for acoustic waves in the n^th^ layer is of the following form:

(12) $$u_{2n}=\left[U_{n1}\text{exp}\left(-ik\beta_{n}x_{3}\right)+U_{n2}\text{exp}\left(ik\beta_{n}x_{3}\right)\right]\cdot \exp\left\{i\left(wt-kx_{1}\right)\right\}\;H_{n+1}<x_{3}<H_{n}$$

where $\beta_{n}=\sqrt{v^{2}/V_{n}^{2}-1}$ and $V_{n}=\sqrt{\mu_{n}/\rho_{n}}$ is the velocity of shear bulk acoustic wave in the waveguide material. *μ_n_* and *ρ_n_* are the shear modulus and mass density of the *n*^th^ (*n* = 1-*N*) layer medium, respectively.

The electric potential in the n^th^ layer can be expressed by:

(13) $$\varphi_{\text{n}}=\left[\Phi_{n1}\exp\left(-kx_{3}\right)+\Phi_{n2}\exp\left(kx_{3}\right)\right]\cdot \exp\left\{i\left(wt-kx_{1}\right)\right\}\;H_{n+1}<x_{3}<H_{n}$$

#### (3) In the air or vacuum

Only electric potential exists in the air or vacuum, and the electric potential will be zero when x_3_→−∞, thus the solution for the electric potential above the guide layer is of the following form:

(14) $$\varphi_{0}=\Phi_{0}\exp\left(-kx_{3}\right)\exp\left\{\text{i}\left(\omega t-kx_{1}\right)\right\}\;x_{3}>H_{1}$$

Then, the values of the undetermined M coefficients in Equation (10) are decided by the boundary conditions at the interface and the surface of the layered structure as following:

1. Mechanical boundary conditions: the particle displacement and stress in the x_3_-direction are continuous on every interface. The normal stress is zero on the surface of the top layer.
2. Electric boundary conditions: the electric potential and electric displacement in the x_3_-direction are continuous on every interface.

Hence, the dispersion equation of Love waves in a multi-waveguides structure can be obtained by the wave equations and the boundary conditions:

(15) $$\frac{T_{2}-X_{N}A_{2}}{T_{1}-X_{N}A_{1}}=\frac{D_{2}-i\bar{{ɛ}_{N}}}{D_{1}-i\bar{{ɛ}_{N}}}$$

where:

(16) $$\{\begin{array}{c} X_{1}=-i\mu_{1}\beta_{1}\tan(kb_{1}h_{1})\\ X_{n}=\mu_{n}\beta_{n}\frac{i\tan\left(k\beta_{n}h_{n}\right)\mu_{n}\beta_{n}+X_{n-1}}{i\tan\left(k\beta_{n}h_{n}\right)X_{n-1}+\mu_{n}\beta_{n}} \end{array},n=2,3,\cdots ,N$$

(17) $$\{\begin{array}{c} T_{1}=\left(c_{44}\beta_{S1}+c_{46}\right)A_{1}+\left(e_{34}\beta_{S1}+e_{14}\right)\\ T_{2}=\left(c_{44}\beta_{S2}+c_{46}\right)A_{2}+\left(e_{34}\beta_{S2}+e_{14}\right) \end{array}$$

(18) $$\{\begin{array}{c} D_{1}=\left(e_{34}\beta_{S1}+e_{36}\right)A_{1}-\left({ɛ}_{33}\beta_{S1}+{ɛ}_{31}\right)\\ D_{2}=\left(e_{34}\beta_{S2}+e_{36}\right)A_{2}-\left({ɛ}_{33}\beta_{S2}+{ɛ}_{31}\right) \end{array}$$

(19) $$\{\begin{array}{c} \bar{{ɛ}_{1}}={ɛ}_{1}\frac{{ɛ}_{1}\tanh\left(kh_{1}\right)+{ɛ}_{0}}{{ɛ}_{0}\tanh\left(kh_{1}\right)+{ɛ}_{1}}\\ \bar{{ɛ}_{n}}={ɛ}_{\text{n}}\frac{{ɛ}_{\text{n}}\tanh\left(kh_{n}\right)+\bar{{ɛ}_{n-1}}}{\bar{{ɛ}_{n-1}}\tanh\left(kh_{n}\right)+{ɛ}_{\text{n}}} \end{array}\;\text{n}=2,3,\ldots \text{N}$$

It is well-known that the material constants of the substrate and the layers vary with different temperature. Thus, the temperature-dependent Love wave phase velocity can be calculated using Equation (15) with temperature dependent material constants. The temperature coefficient of frequency (*Tcf*) can be defined as:

(20) $$\text{Tcf}=\frac{v_{35}-v_{15}}{20v_{25}}-\alpha$$

where *α* is coefficient of thermal expansion in the substrate, *v_15_*, *v_25_*, *v_35_* refer to Love wave phase velocity at 15 °C, 25 °C, 35 °C.

The temperature dependent material constants is approximated by a second-order function:

(21) $$X=X_{0}\;[1+\alpha_{1}\left(T-T_{0}\right)+\alpha_{2}{\left(T-T_{0}\right)}^{2}]$$

where *X* refers to material constants at various temperature of *T*, *X*_0_ is the material constants at room temperature, *T*_0_, α_1_ and α_2_ are the first- and second-order temperature coefficients of the material constants, respectively. Table 2 lists the material constants used in this paper.

### 2.2. Numerical Results and Discussion

Here, we just consider the Love wave propagating along the multi-guiding layer on ST-90°X quartz, and the SiO_2_/SU-8 act as the wave guide layers, and the temperature-dependent mechanical parameters of the guiding layers and substrates like the density, elastic constants, piezoelectricity and dielectric constants of the above media are as listed in Table 2.

**Figure 3.**
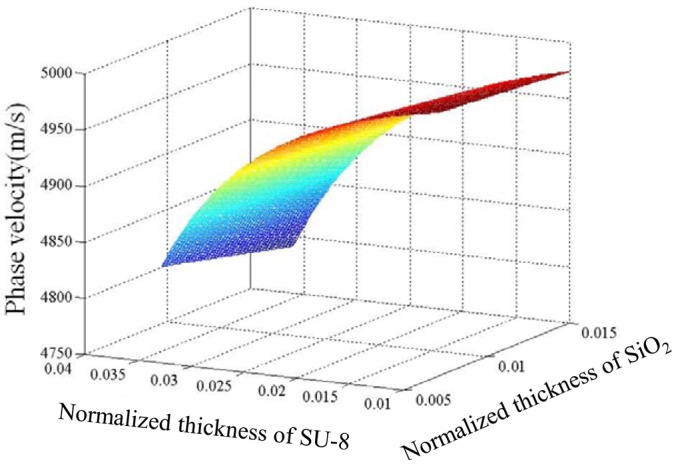
Phase velocity versus normalized layer thickness in a layered structure of ST-90°X quartz/SiO_2_/SU-8 at room temperature of 25 °C.

Based on the theoretical formulas mentioned above, and the mechanical parameters of the substrate and guide layers listed in Table 2, the dispersion properties of a Love wave in a layered structure can be analyzed, and the phase velocity of the Love wave depending on the guiding layer thickness at room temperature of 25 °C is as depicted in Figure 3. Only the fundamental Love wave mode exists under the thickness of waveguides we are interested in. From the calculated results, the Love wave velocity decreases with increases of the thickness of the guide layers, and the effect on the Love wave velocity from the SU-8 thickness was more obvious over the SiO_2_ guide layer.

**Figure 4.**
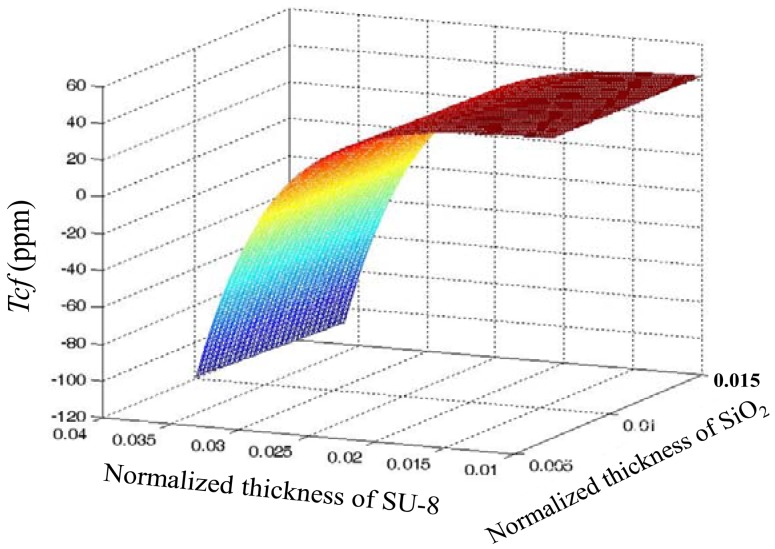
Calculated *Tcf versus* normalized layer thickness.

Figure 4 shows calculated *Tcf* versus normalized thickness of the guide layers in a layered structure of ST-90°X quartz/SiO_2_/SU-8 using Equation (20). The *Tcf* value is influenced by SU-8 thickness far more significantly than by that of SiO_2_. Considering the technical difficulties of thick SiO_2_ film deposition, a thin SiO_2_ coating is advised, so, from Figure 3, we can see that when the thickness of SiO_2_ is 0.2 μm and SU-8 0.826 μm are applied; almost zero *Tcf* of the device may be achieved.

## 3. Experiments

### 3.1. Fabrication of the Love Wave Device

The Love wave delay lines with a SiO_2_/SU-8 guiding layer were developed on a ST-90°X quartz substrate. First the SH-SAW delay line on ST-90°X quartz with two photolithographically defined Al (250 nm) transducers separated by a path length (transducer center separation) of 2.5 mm was fabricated. The two transducers consist of 120 and 40 finger-pairs interdigitated electrodes, with periodicity of 28 μm for an operation frequency of 175 MHz. A single phase unidirectional transducer (SPUDT) was used to structure the transducers to reduce the low insertion loss [21]. Then, SiO_2_ thin film was evaporated on the fresh surface of the SH-SAW device by the lift-off technique at room temperature with a sacrificial layer of photoresist 5214. The thickness of SiO_2_ was set to 0.21 μm according to the theoretical prediction, and monitored by an Alpha-Step IQ profiler. After the SiO_2_ coating, the SU-8 2050 with various thicknesses produced by the Microchem Company was deposited onto the SiO_2_ surface by spin coating. The design parameters of the fabricated devices are as listed in Table 3.

**Figure 5.**
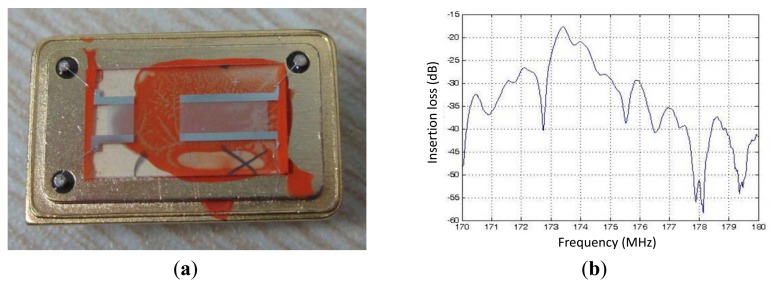
(**a**) the fabricated Love wave device; (**b**) the measured frequency response (S_21_) of the fabricated device.

Figure 5(a) shows the fabricated Love wave devices with size of 9 × 3 mm, and also, the frequency response (S_21_) of the fabricated Love wave device with SU-8 thickness of 0.8 μm, tagged as device #1, which was characterized by a network analyzer (Advantest R3765), as shown in Figure 5(b). Insertion loss of ∼17dB was observed at the frequency of 173.38 MHz.

### 3.2. Experimental Setup

**Figure 6.**
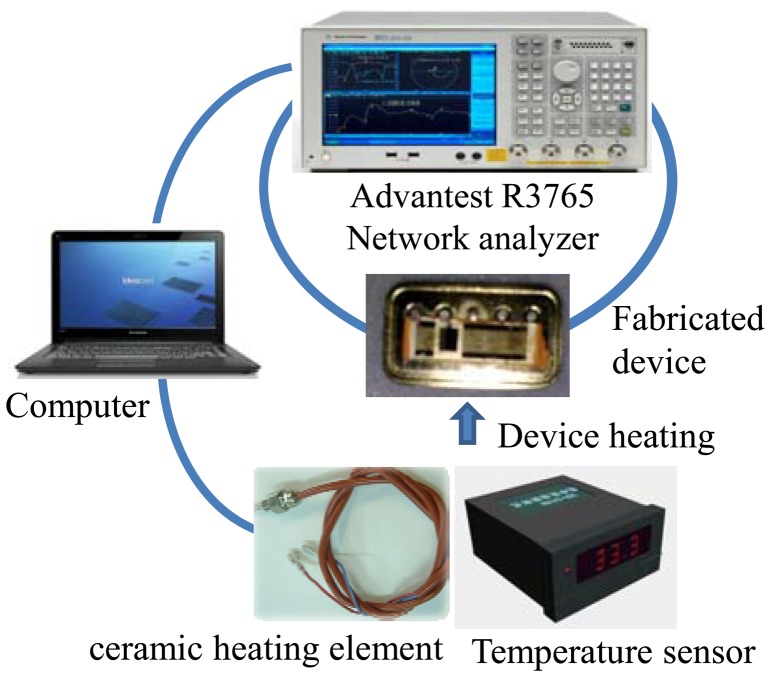
The temperature stability measurement setup of the fabricated devices.

The temperature properties of the fabricated Love wave devices were evaluated experimentally. The measurement setup, which includes the network analyzer, temperature sensor, ceramic heating element and computer, is depicted in Figure 6. The fabricated Love wave delay lines with different SU-8 thicknesses listed in Table 2 were heated by the ceramic heating element, and the network analyzer was used to monitor and recording the frequency shift depending on the applied temperature change. The temperature change of the Love wave device is also read out by the temperature sensor connected to pt100 posted at the bottom of the Love wave device. Both frequency and temperature are acquired in real time by the PC connected to network analyzer and temperature sensor.

### 3.3. Experimental Results and Discussion

**Figure 7.**
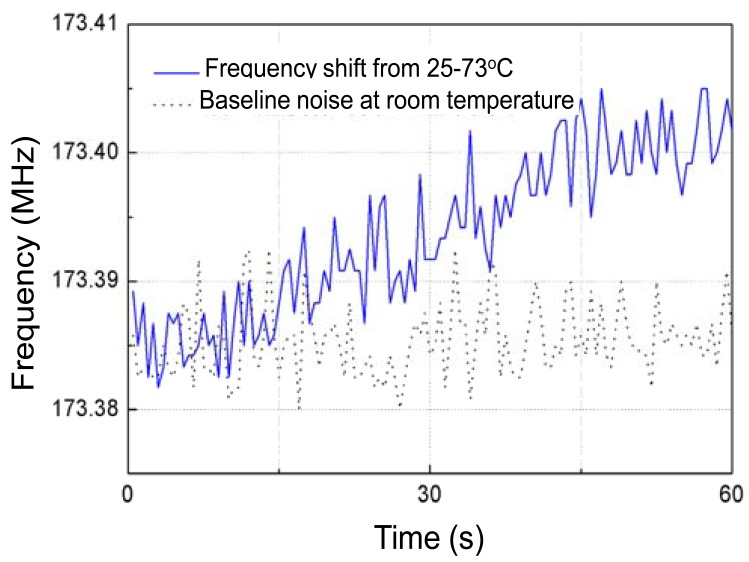
The measured baseline noise and time-dependent frequency response of the device #3.

Prior to measurement, the baseline noise of the Love wave device in testing time of 60 s was tested at room temperature (25 °C). It is shown as the dotted line in Figure 7. A frequency shift of ∼10 kHz was observed.

**Figure 8.**
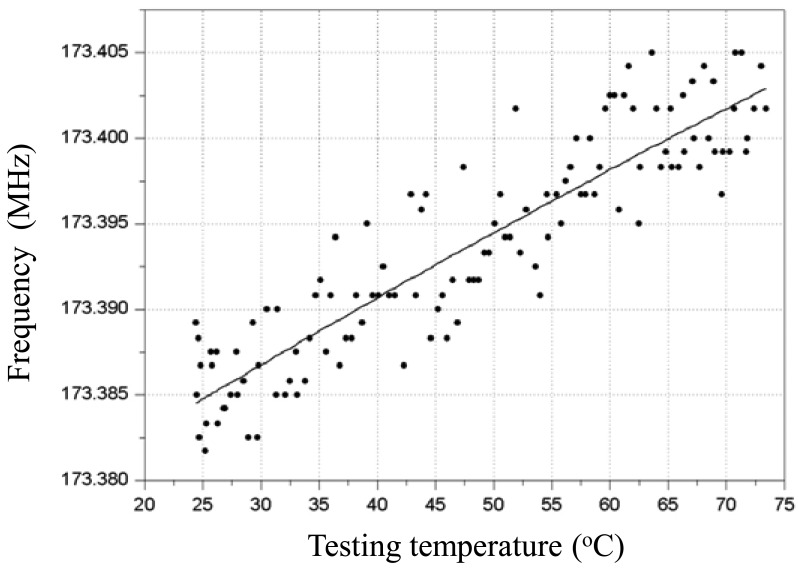
The time-dependent frequency response of the device #3.

**Figure 9.**
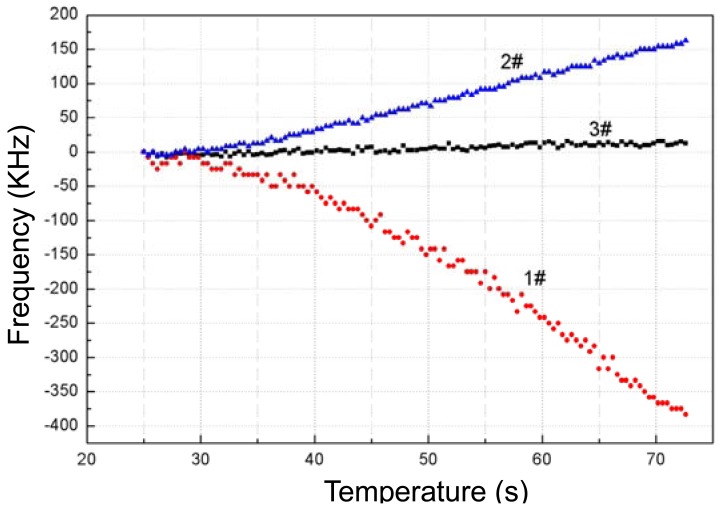
Measured temperature-dependent frequency shift of the devices #1, #2, and #3.

**Figure 10.**
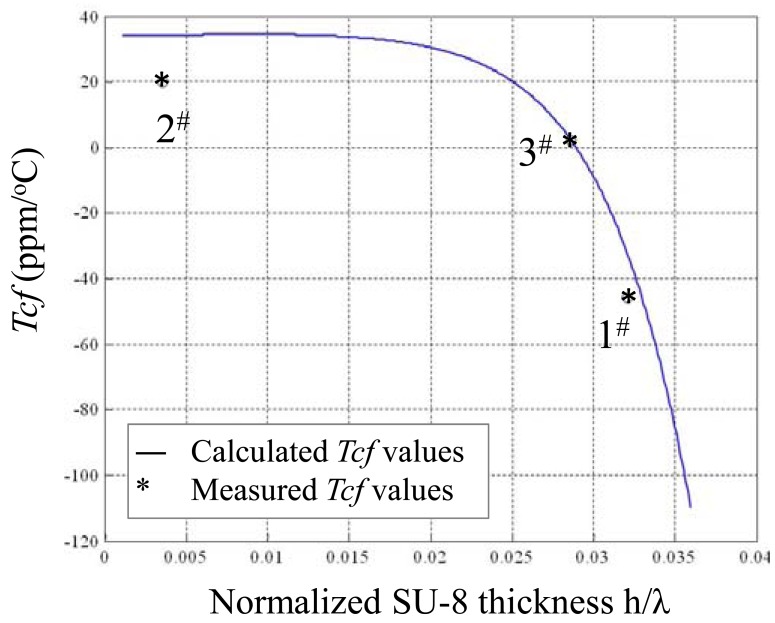
*Tcf* comparison between the experimental results and theoretical calculations.

Then, the frequency response depending on temperature variation from 25 °C to 73 °C of the fabricated Love wave delay line (device #3) was measured as shown in Figure 7, and a frequency shift of around 0.018MHz with a temperature increase of 48 °C was observed. The binomial fitting of the measured frequency shift in temperature range of 25 °C∼73 °C was plotted in Figure 8. A very low *Tcf* of 2.16 ppm/°C (*Tcf* = 0.018/48/173.38 × 10^6^) was deduced experimentally, and it is far more less than typical Love wave structure (ST-90°X quartz/SiO_2_) of 27–31 ppm/°C [8]. Also, due to the insertion loss effect due to strain, and the stress effect between the SU-8 and SiO_2_ layers as temperature increases, another insertion loss of ∼5 dB was induced when the temperature was increased to 73 °C. The temperature properties of another two devices (device 1# and 2#) in the temperature range of 25 °C∼73 °C were also tested as shown in Figure 9, and the corresponding *Tcf* values for the device 1# and 2# sre depicted in Figure 10.

The measured data agrees well with the theoretical analysis as above. When thicker SU-8 was applied, the frequency decreases quickly due to the larger viscoelastic nature of the polymer itself. It means that the optimal thicknesses of the multi-guide layers of SiO_2_/SU-8 are determined as 0.21 μm/0.8 μm. From these promising results, we conclude that the temperature stability of a Love wave device can be improved effectively by controlling the wave guide layer thickness.

## 4. Conclusions

The temperature effect on Love wave propagation along a layered structure was analyzed theoretically. The Love wave device with multi-guide layers of SU-8/SiO_2_/ST-90°X quartz and a very low temperature coefficient of frequency (*Tcf*) of 2.16 ppm/°C was implemented based on establishment of the corresponding theoretical model. The optimal guide layer thicknesses were determined, leading to excellent temperature stability. The theoretical model was confirmed by the experiments.

## Figures and Tables

**Table 1.** Physical parameters of the materials for guide layer and substrate.

**Materials**	**Shear Velocity (m/s)**	**Density (kg/m^3^)**	**Polarity of Temperature Coefficient**

64efficien_3_	4,608	4,700	-
41efficien_3_	4,792		

3600ficien_3_	4,202	7,450	-

ST-90ic quartz	5,060	2,650	+
36artquartz	5,100		

SiO_2_	2,850	2,650	+

PMMA	1,100	1,140	-

SU-8	1,810	1,190	-

**Table 2.** The temperature-dependent parameters of the substrate and guide layers [11,19,20].

**Materials**	**Parameters**	***X****_0_* **at reference temperature of *T****_0_*	**α_1_ (10**^−^**^4^)**	**α_2_ (10**^−^**^7^)**

Quartz	c_11_(10^11^ N/m^2^)	0.8674	−0.443	−4.07
T_0_ = 20 °C	c_12_(10^11^ N/m^2^)	0.0699	−29.3	72.45
	c_13_(10^11^ N/m^2^)	0.1191	−4.92	−5.96
	c_14_(10^11^ N/m^2^)	−0.1791	0.98	−0.13
	c_33_(10^11^ N/m^2^)	1.072	−1.88	−14.12
	c_44_(10^11^ N/m^2^)	0.5794	−1.72	−2.25
	c_66_(10^11^ N/m^2^)	0.3988	1.8	2.01
	ρ(10^3^ kg/m^3^)	2.65	−0.3492	−0.159
	e_11_(C/m^2^)	0.171		
	e_14_(C/m^2^)	0.0403		
	ε_11_(10^−11^ F/m^2^)	3.997		
	ε_33_(10^−11^ F/m^2^)	4.103		
	α_11_(10^−6^ /°C)	13.71		
	α_33_(10^−6^ /°C)	7.48		

SiO_2_	ρ(10^3^ kg/m^3^)	2.2		
T_0_ = 25 °C	μ(10^11^ N/m^2^)	0.3121	1.4553	
	ε_11_(10^−11^ F/m^2^)	3.8265	0.2628	

SU-8	ρ(10^3^ kg/m^3^)	1.2152	−4.5142	1.3302
T_0_ = 25 °C	μ(10^11^ N/m^2^)	0.011769		83.3139
	ε_11_(10^−11^ F/m^2^)	2.6563	−37.757	

**Table 3.** Design parameters of the fabricated devices.

**Device #**	**SiO_2_ Thickness (μm)**	**SU-8 Thickness (μm)/(h/λ)**
1	0.21	0.9/0.032
2	0.21	0.1/0.0035
3	0.21	0.8/0.028
